# 5-Nitro-1-nonyl-1*H*-benzimidazol-2(3*H*)-one

**DOI:** 10.1107/S1600536811005654

**Published:** 2011-02-19

**Authors:** Younes Ouzidan, Youssef Kandri Rodi, El Mokhtar Essassi, Lahcen El Ammari, Frank R. Fronczek, Ramaiyer Venkatraman

**Affiliations:** a Laboratoire de Chimie Organique Appliquée, Université Sidi Mohamed, Ben Abdallah, Faculté des Sciences et Techniques, Route d’Immouzzer, BP 2202 Fès, Morocco; b Laboratoire de Chimie Organique Hétérocyclique, Pôle de Compétences, Pharmacochimie, Av Ibn Battouta, BP 1014, Faculté des Sciences, Université Mohammed V-Agdal, Rabat, Morocco; c Laboratoire de Chimie du Solide Appliquée, Faculté des Sciences, Université Mohammed V-Agdal, Avenue Ibn Battouta, BP 1014, Rabat, Morocco; d Department of Chemistry, Louisiana State University, Baton Rouge, LA 70803, USA; e Department of Chemistry and Biochemistry, Jackson State University, Jackson, MS 39217, USA

## Abstract

In the title mol­ecule, C_16_H_23_N_3_O_3_, the dihedral angle between the benzimidazole and nitro group planes is 5.34 (9)° and the dihedral angle between the benzimidazole and aliphatic chain mean planes is 73.23 (5)°. The C—C—C—C torsion angles (about 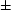
176°) of the nonyl group indicate an all-anti­periplanar conformation. In the crystal, adjacent mol­ecules are linked by pairs of N—H⋯O hydrogen bonds into inversion dimers. These mol­ecules are further connected through C—H⋯O inter­actions, building tapes parallel to (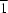
22).

## Related literature

For background to the pharmacological and biochemical properties of benzimidazolo­nes, see: Gbadamassi *et al.* (1988 ▶); Singh *et al.* (2000 ▶); Derand *et al.* (2003 ▶); Badarau *et al.* (2009 ▶). For similar structures, see: Saber *et al.* (2010 ▶); Ouzidan *et al.* (2011 ▶).
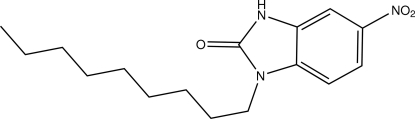


## Experimental

### 

#### Crystal data

C_16_H_23_N_3_O_3_
*M* *_r_* = 305.37
Triclinic, 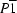

*a* = 5.483 (1) Å
*b* = 10.2092 (15) Å
*c* = 14.746 (3) Å
α = 74.275 (9)°
β = 79.727 (6)°
γ = 83.410 (8)°
*V* = 779.9 (2) Å^3^
*Z* = 2
Mo *K*α radiation
μ = 0.09 mm^−1^
*T* = 90 K
0.35 × 0.27 × 0.22 mm

#### Data collection

Nonius KappaCCD diffractometer
21087 measured reflections
6349 independent reflections
5183 reflections with *I* > 2σ(*I*)
*R*_int_ = 0.023

#### Refinement

*R*[*F* ^2^ > 2σ(*F* ^2^)] = 0.040
*wR*(*F* ^2^) = 0.113
*S* = 1.03
6349 reflections
201 parameters
H-atom parameters constrained
Δρ_max_ = 0.45 e Å^−3^
Δρ_min_ = −0.28 e Å^−3^

### 

Data collection: *COLLECT* (Nonius, 2000 ▶); cell refinement: *SCALEPACK* (Otwinowski & Minor, 1997 ▶); data reduction: *DENZO* (Otwinowski & Minor, 1997 ▶) and *SCALEPACK*; program(s) used to solve structure: *SIR97* (Altomare *et al.*, 1999 ▶); program(s) used to refine structure: *SHELXL97* (Sheldrick, 2008 ▶); molecular graphics: *ORTEP-3 for Windows* (Farrugia, 1997 ▶) and *PLATON* (Spek, 2009 ▶); software used to prepare material for publication: *SHELXL97*.

## Supplementary Material

Crystal structure: contains datablocks I, global. DOI: 10.1107/S1600536811005654/gk2342sup1.cif

Structure factors: contains datablocks I. DOI: 10.1107/S1600536811005654/gk2342Isup2.hkl

Additional supplementary materials:  crystallographic information; 3D view; checkCIF report

## Figures and Tables

**Table 1:** Hydrogen-bond geometry (Å, °)

*D*—H⋯*A*	*D*—H	H⋯*A*	*D*⋯*A*	*D*—H⋯*A*
N1—H1⋯O1^i^	0.88	1.89	2.7651 (9)	170
C6—H6⋯O3^ii^	0.95	2.58	3.3139 (11)	134

Symmetry codes: (i) 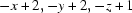; (ii) 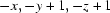.

**Fig. 1.**
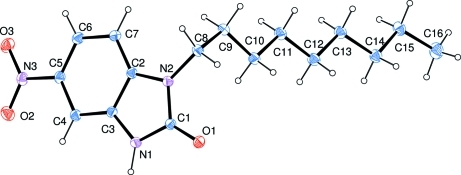
: Molecular structure of the title compound with displacement ellipsoids drawn at the 50% probability level. H atoms are represented as small circles.

**Fig. 2.**
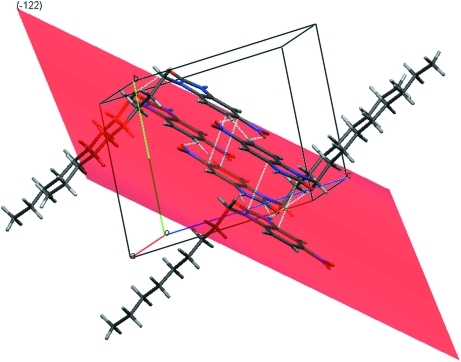
Partial packing view of the title compound, showing tapes in the (-1 2 2) plane, built up from molecules linked through N—H···O hydrogen bonds and intermolecular C–H···O contacts (dashed lines).

## supplementary crystallographic information

### Comment

Benzimidazoles are useful intermediates/subunits for the development of molecules of pharmaceutical or biological interest (Gbadamassi *et al.,* 1988). Benzimidazolone and its derivatives are also an important class of bioactive molecules in the field of drugs and pharmaceuticals (Derand *et al.*, 2003). They found potential applications in diverse therapeutic areas including, anti-hypertensives and anti-virals (Badarau *et al.*, 2009; Singh *et al.*, 2000). The structural studies of benzimidazolone, linked to an isopropenyl and nonyl group respectively, have been published by Saber *et al.* (2010)and Ouzidan *et al.* (2011).

The 5-nitro-1-nonyl-1*H*-benzimidazol-2(3*H*)-one molecule structure is built up from two fused six-and five-membered rings linked to C_9_H_19_ chain as shown in Fig.1. The aliphatic chain has all-antiperiplanar (all-*trans*) conformation. Furthermore, the fused-ring system and the nitro group are almost planar, with a maximum deviation of 0.0414 (8) Å and 0.0250 (7) Å for C4 and N1 respectively. The dihedral angle between the two rings and nitro group planes is 5.34 (9)°. The torsion angles C1 N2 C8 C9 and C13 C14 C15 C16 are 113.66 (8)° and 177.26 (7)° respectively.

In the crystal, adjacent molecules are linked by pairs of N—H···O hydrogen bonds into inversion dimers. These molecules are further connected through C—H···O hydrogen bonds into a tape parallel to the (-1 2 2) plane, as schown in Fig. 2 and Table 1.

### Experimental

To 5-nitro-1*H*-benzoimidazol-2(3*H*)-one (0.2 g, 1.1 mmol), potassium carbonate (0.30 g, 2.2 mmol) and tetra-n-butylammonium bromide (0.07 g, 0.2 mmol) in DMF (15 ml) was added 1-bromononane (0.43 ml, 2.2 mmol). Stirring was continued at room temperature for 6 h. The salt was removed by filtration and the filtrate concentrated under reduced pressure. The residue was separated by chromatography on a column of silica gel with ethyl acetate/hexane (1/2) as eluent. Colorless needle-shaped crystals were isolated when the solvent was allowed to evaporate [(m.p. 392–394 K (ethanol)].

### Refinement

H atoms were located in a difference map and treated as riding with C—H = 0.99 Å, 0.98, Å, 0.95 Å, and 0.88 Å for –CH_2_–, –CH_3_, aromatic CH and NH respectively. All H atoms with *U*_iso_(H) = 1.2 *U*_eq_ (aromatic, methylene, N) and *U*_iso_(H) = 1.5 *U*_eq_(methyl).

### Crystal data

**Table:**

C_16_H_23_N_3_O_3_	*Z* = 2
*M**_r_* = 305.37	*F*(000) = 328
Triclinic, *P*1	*D*_x_ = 1.300 Mg m^−^^3^
Hall symbol: -P 1	Mo *K*α radiation, λ = 0.71073 Å
*a* = 5.483 (1) Å	Cell parameters from 5537 reflections
*b* = 10.2092 (15) Å	θ = 2.5–34.9°
*c* = 14.746 (3) Å	µ = 0.09 mm^−^^1^
α = 74.275 (9)°	*T* = 90 K
β = 79.727 (6)°	Needle, colourless
γ = 83.410 (8)°	0.35 × 0.27 × 0.22 mm
*V* = 779.9 (2) Å^3^	

### Data collection

**Table:**

Nonius KappaCCD diffractometer	5183 reflections with *I* > 2σ(*I*)
Radiation source: fine-focus sealed tube	*R*_int_ = 0.023
graphite	θ_max_ = 34.9°, θ_min_ = 2.8°
ω and φ scans	*h* = −8→8
21087 measured reflections	*k* = −15→16
6349 independent reflections	*l* = −23→22

### Refinement

**Table:**

Refinement on *F*^2^	Secondary atom site location: difference Fourier map
Least-squares matrix: full	Hydrogen site location: inferred from neighbouring sites
*R*[*F*^2^ > 2σ(*F*^2^)] = 0.040	H-atom parameters constrained
*wR*(*F*^2^) = 0.113	*w* = 1/[σ^2^(*F*_o_^2^) + (0.0578*P*)^2^ + 0.1462*P*] where *P* = (*F*_o_^2^ + 2*F*_c_^2^)/3
*S* = 1.03	(Δ/σ)_max_ = 0.001
6349 reflections	Δρ_max_ = 0.45 e Å^−^^3^
201 parameters	Δρ_min_ = −0.28 e Å^−^^3^
0 restraints	Extinction correction: *SHELXL97* (Sheldrick, 2008), Fc^*^=kFc[1+0.001xFc^2^λ^3^/sin(2θ)]^-1/4^
Primary atom site location: structure-invariant direct methods	Extinction coefficient: 0.023 (5)

### Special details

**Table:**

Geometry. All s.u.'s (except the s.u. in the dihedral angle between two l.s. planes) are estimated using the full covariance matrix. The cell s.u.'s are taken into account individually in the estimation of s.u.'s in distances, angles and torsion angles; correlations between s.u.'s in cell parameters are only used when they are defined by crystal symmetry. An approximate (isotropic) treatment of cell s.u.'s is used for estimating s.u.'s involving l.s. planes.
Refinement. Refinement of *F*^2^ against ALL reflections. The weighted *R*-factor *wR* and goodness of fit *S* are based on *F*^2^, conventional *R*-factors *R* are based on *F*, with *F* set to zero for negative *F*^2^. The threshold expression of *F*^2^ > σ(*F*^2^) is used only for calculating *R*-factors(gt) *etc*. and is not relevant to the choice of reflections for refinement. *R*-factors based on *F*^2^ are statistically about twice as large as those based on *F*, and *R*- factors based on ALL data will be even larger.

### Fractional atomic coordinates and isotropic or equivalent isotropic displacement parameters (Å^2^)

**Table:**

	*x*	*y*	*z*	*U*_iso_*/*U*_eq_	
O1	0.92795 (11)	1.07035 (6)	0.37556 (4)	0.01618 (12)	
O2	0.40529 (12)	0.45052 (6)	0.71989 (4)	0.02109 (13)	
O3	0.12666 (12)	0.41865 (6)	0.64166 (5)	0.02143 (13)	
N1	0.78463 (12)	0.88673 (7)	0.50035 (5)	0.01369 (12)	
H1	0.8751	0.8904	0.5431	0.016*	
N2	0.61923 (12)	0.93607 (7)	0.36748 (4)	0.01292 (12)	
N3	0.29292 (13)	0.48382 (7)	0.65097 (5)	0.01591 (13)	
C1	0.79248 (13)	0.97452 (8)	0.41127 (5)	0.01294 (13)	
C2	0.50448 (13)	0.82444 (7)	0.42883 (5)	0.01255 (13)	
C3	0.61268 (13)	0.79137 (8)	0.51286 (5)	0.01240 (13)	
C4	0.54726 (13)	0.68061 (8)	0.58770 (5)	0.01379 (13)	
H4	0.6229	0.6565	0.6437	0.017*	
C5	0.36219 (13)	0.60623 (7)	0.57568 (5)	0.01384 (13)	
C6	0.24677 (14)	0.63920 (8)	0.49481 (6)	0.01500 (13)	
H6	0.1193	0.5860	0.4912	0.018*	
C7	0.31799 (14)	0.75013 (8)	0.41917 (5)	0.01431 (13)	
H7	0.2421	0.7740	0.3632	0.017*	
C8	0.56136 (14)	1.00911 (8)	0.27295 (5)	0.01453 (13)	
H8A	0.6475	1.0948	0.2508	0.017*	
H8B	0.3804	1.0338	0.2778	0.017*	
C9	0.63787 (14)	0.92606 (8)	0.19926 (5)	0.01496 (13)	
H9A	0.5714	0.9758	0.1402	0.018*	
H9B	0.5589	0.8382	0.2237	0.018*	
C10	0.91760 (14)	0.89612 (8)	0.17380 (5)	0.01536 (14)	
H10A	0.9848	0.8397	0.2311	0.018*	
H10B	1.0005	0.9829	0.1522	0.018*	
C11	0.97314 (15)	0.82089 (8)	0.09493 (6)	0.01648 (14)	
H11A	0.8959	0.7325	0.1186	0.020*	
H11B	0.8936	0.8751	0.0400	0.020*	
C12	1.24889 (15)	0.79376 (8)	0.06003 (6)	0.01714 (14)	
H12A	1.3281	0.7349	0.1137	0.021*	
H12B	1.3293	0.8813	0.0386	0.021*	
C13	1.29159 (14)	0.72462 (8)	−0.02204 (6)	0.01650 (14)	
H13A	1.2188	0.6349	0.0010	0.020*	
H13B	1.2018	0.7809	−0.0735	0.020*	
C14	1.56428 (15)	0.70296 (8)	−0.06374 (6)	0.01707 (14)	
H14A	1.6346	0.7928	−0.0918	0.020*	
H14B	1.6575	0.6522	−0.0118	0.020*	
C15	1.59873 (15)	0.62433 (9)	−0.14015 (6)	0.01812 (15)	
H15A	1.5350	0.5330	−0.1111	0.022*	
H15B	1.4981	0.6728	−0.1903	0.022*	
C16	1.86819 (17)	0.60708 (10)	−0.18635 (7)	0.02506 (18)	
H16A	1.9271	0.6967	−0.2215	0.038*	
H16B	1.8795	0.5492	−0.2304	0.038*	
H16C	1.9712	0.5643	−0.1368	0.038*	

### Atomic displacement parameters (Å^2^)

**Table:**

	*U*^11^	*U*^22^	*U*^33^	*U*^12^	*U*^13^	*U*^23^
O1	0.0171 (2)	0.0163 (3)	0.0153 (2)	−0.00631 (19)	−0.00140 (19)	−0.0029 (2)
O2	0.0265 (3)	0.0195 (3)	0.0162 (3)	−0.0041 (2)	−0.0049 (2)	−0.0008 (2)
O3	0.0224 (3)	0.0188 (3)	0.0235 (3)	−0.0096 (2)	−0.0006 (2)	−0.0048 (2)
N1	0.0147 (3)	0.0147 (3)	0.0125 (3)	−0.0040 (2)	−0.0026 (2)	−0.0035 (2)
N2	0.0138 (3)	0.0137 (3)	0.0117 (3)	−0.0029 (2)	−0.0018 (2)	−0.0032 (2)
N3	0.0177 (3)	0.0143 (3)	0.0153 (3)	−0.0031 (2)	0.0006 (2)	−0.0044 (2)
C1	0.0130 (3)	0.0138 (3)	0.0126 (3)	−0.0014 (2)	−0.0010 (2)	−0.0048 (2)
C2	0.0120 (3)	0.0132 (3)	0.0126 (3)	−0.0014 (2)	−0.0005 (2)	−0.0043 (2)
C3	0.0119 (3)	0.0133 (3)	0.0130 (3)	−0.0020 (2)	−0.0009 (2)	−0.0052 (2)
C4	0.0143 (3)	0.0145 (3)	0.0128 (3)	−0.0020 (2)	−0.0011 (2)	−0.0041 (2)
C5	0.0147 (3)	0.0123 (3)	0.0141 (3)	−0.0024 (2)	0.0000 (2)	−0.0034 (2)
C6	0.0140 (3)	0.0150 (3)	0.0168 (3)	−0.0028 (2)	−0.0017 (2)	−0.0051 (3)
C7	0.0132 (3)	0.0159 (3)	0.0148 (3)	−0.0020 (2)	−0.0030 (2)	−0.0046 (2)
C8	0.0158 (3)	0.0147 (3)	0.0125 (3)	0.0003 (2)	−0.0026 (2)	−0.0028 (2)
C9	0.0157 (3)	0.0172 (3)	0.0125 (3)	−0.0017 (2)	−0.0026 (2)	−0.0043 (2)
C10	0.0163 (3)	0.0171 (3)	0.0135 (3)	−0.0011 (2)	−0.0024 (2)	−0.0053 (3)
C11	0.0173 (3)	0.0189 (3)	0.0142 (3)	−0.0006 (3)	−0.0017 (2)	−0.0065 (3)
C12	0.0179 (3)	0.0196 (4)	0.0153 (3)	−0.0003 (3)	−0.0029 (3)	−0.0071 (3)
C13	0.0168 (3)	0.0184 (3)	0.0150 (3)	−0.0001 (3)	−0.0020 (2)	−0.0063 (3)
C14	0.0173 (3)	0.0181 (3)	0.0157 (3)	−0.0004 (3)	−0.0015 (3)	−0.0053 (3)
C15	0.0200 (3)	0.0183 (4)	0.0157 (3)	0.0012 (3)	−0.0015 (3)	−0.0056 (3)
C16	0.0217 (4)	0.0302 (5)	0.0221 (4)	0.0022 (3)	0.0013 (3)	−0.0094 (3)

### Geometric parameters (Å, °)

**Table:**

O1—C1	1.2387 (9)	C9—H9A	0.9900
O2—N3	1.2313 (9)	C9—H9B	0.9900
O3—N3	1.2352 (9)	C10—C11	1.5297 (11)
N1—C1	1.3715 (10)	C10—H10A	0.9900
N1—C3	1.3864 (9)	C10—H10B	0.9900
N1—H1	0.8800	C11—C12	1.5268 (11)
N2—C1	1.3838 (10)	C11—H11A	0.9900
N2—C2	1.3842 (10)	C11—H11B	0.9900
N2—C8	1.4628 (10)	C12—C13	1.5303 (11)
N3—C5	1.4646 (10)	C12—H12A	0.9900
C2—C7	1.3893 (10)	C12—H12B	0.9900
C2—C3	1.4109 (10)	C13—C14	1.5272 (11)
C3—C4	1.3787 (11)	C13—H13A	0.9900
C4—C5	1.3970 (11)	C13—H13B	0.9900
C4—H4	0.9500	C14—C15	1.5263 (11)
C5—C6	1.3924 (11)	C14—H14A	0.9900
C6—C7	1.3930 (11)	C14—H14B	0.9900
C6—H6	0.9500	C15—C16	1.5246 (12)
C7—H7	0.9500	C15—H15A	0.9900
C8—C9	1.5266 (11)	C15—H15B	0.9900
C8—H8A	0.9900	C16—H16A	0.9800
C8—H8B	0.9900	C16—H16B	0.9800
C9—C10	1.5284 (11)	C16—H16C	0.9800
			
C1—N1—C3	109.78 (6)	C9—C10—C11	110.76 (6)
C1—N1—H1	125.1	C9—C10—H10A	109.5
C3—N1—H1	125.1	C11—C10—H10A	109.5
C1—N2—C2	109.39 (6)	C9—C10—H10B	109.5
C1—N2—C8	124.01 (6)	C11—C10—H10B	109.5
C2—N2—C8	126.52 (6)	H10A—C10—H10B	108.1
O2—N3—O3	123.41 (7)	C12—C11—C10	114.85 (6)
O2—N3—C5	118.20 (7)	C12—C11—H11A	108.6
O3—N3—C5	118.39 (7)	C10—C11—H11A	108.6
O1—C1—N1	127.21 (7)	C12—C11—H11B	108.6
O1—C1—N2	125.74 (7)	C10—C11—H11B	108.6
N1—C1—N2	107.04 (6)	H11A—C11—H11B	107.5
N2—C2—C7	131.59 (7)	C11—C12—C13	112.22 (6)
N2—C2—C3	106.99 (6)	C11—C12—H12A	109.2
C7—C2—C3	121.42 (7)	C13—C12—H12A	109.2
C4—C3—N1	131.24 (7)	C11—C12—H12B	109.2
C4—C3—C2	121.98 (7)	C13—C12—H12B	109.2
N1—C3—C2	106.77 (6)	H12A—C12—H12B	107.9
C3—C4—C5	115.49 (7)	C14—C13—C12	114.38 (7)
C3—C4—H4	122.3	C14—C13—H13A	108.7
C5—C4—H4	122.3	C12—C13—H13A	108.7
C6—C5—C4	123.69 (7)	C14—C13—H13B	108.7
C6—C5—N3	118.32 (7)	C12—C13—H13B	108.7
C4—C5—N3	117.95 (7)	H13A—C13—H13B	107.6
C5—C6—C7	120.08 (7)	C15—C14—C13	112.45 (7)
C5—C6—H6	120.0	C15—C14—H14A	109.1
C7—C6—H6	120.0	C13—C14—H14A	109.1
C2—C7—C6	117.29 (7)	C15—C14—H14B	109.1
C2—C7—H7	121.4	C13—C14—H14B	109.1
C6—C7—H7	121.4	H14A—C14—H14B	107.8
N2—C8—C9	113.04 (6)	C16—C15—C14	113.56 (7)
N2—C8—H8A	109.0	C16—C15—H15A	108.9
C9—C8—H8A	109.0	C14—C15—H15A	108.9
N2—C8—H8B	109.0	C16—C15—H15B	108.9
C9—C8—H8B	109.0	C14—C15—H15B	108.9
H8A—C8—H8B	107.8	H15A—C15—H15B	107.7
C8—C9—C10	115.31 (6)	C15—C16—H16A	109.5
C8—C9—H9A	108.4	C15—C16—H16B	109.5
C10—C9—H9A	108.4	H16A—C16—H16B	109.5
C8—C9—H9B	108.4	C15—C16—H16C	109.5
C10—C9—H9B	108.4	H16A—C16—H16C	109.5
H9A—C9—H9B	107.5	H16B—C16—H16C	109.5
			
C3—N1—C1—O1	−178.59 (7)	C3—C4—C5—N3	177.28 (6)
C3—N1—C1—N2	1.29 (8)	O2—N3—C5—C6	175.91 (7)
C2—N2—C1—O1	179.64 (7)	O3—N3—C5—C6	−3.41 (11)
C8—N2—C1—O1	−3.45 (12)	O2—N3—C5—C4	−1.82 (10)
C2—N2—C1—N1	−0.24 (8)	O3—N3—C5—C4	178.86 (7)
C8—N2—C1—N1	176.67 (6)	C4—C5—C6—C7	1.40 (12)
C1—N2—C2—C7	178.96 (8)	N3—C5—C6—C7	−176.19 (7)
C8—N2—C2—C7	2.15 (13)	N2—C2—C7—C6	178.47 (7)
C1—N2—C2—C3	−0.87 (8)	C3—C2—C7—C6	−1.72 (11)
C8—N2—C2—C3	−177.68 (6)	C5—C6—C7—C2	−0.33 (11)
C1—N1—C3—C4	176.91 (8)	C1—N2—C8—C9	113.65 (8)
C1—N1—C3—C2	−1.82 (8)	C2—N2—C8—C9	−69.97 (9)
N2—C2—C3—C4	−177.25 (7)	N2—C8—C9—C10	−66.44 (9)
C7—C2—C3—C4	2.90 (11)	C8—C9—C10—C11	−176.26 (6)
N2—C2—C3—N1	1.62 (8)	C9—C10—C11—C12	176.47 (7)
C7—C2—C3—N1	−178.23 (7)	C10—C11—C12—C13	−177.19 (7)
N1—C3—C4—C5	179.65 (7)	C11—C12—C13—C14	176.46 (7)
C2—C3—C4—C5	−1.79 (11)	C12—C13—C14—C15	175.71 (7)
C3—C4—C5—C6	−0.32 (11)	C13—C14—C15—C16	177.26 (7)

### Hydrogen-bond geometry (Å, °)

**Table:**

*D*—H···*A*	*D*—H	H···*A*	*D*···*A*	*D*—H···*A*
N1—H1···O1^i^	0.88	1.89	2.7651 (9)	170
C6—H6···O3^ii^	0.95	2.58	3.3139 (11)	134

Symmetry codes: (i) −*x*+2, −*y*+2, −*z*+1; (ii) −*x*, −*y*+1, −*z*+1.
